# Infant feeding pattern in the first six months of age in USA: a follow-up study

**DOI:** 10.1186/s13006-017-0139-4

**Published:** 2017-12-02

**Authors:** Wilfried Karmaus, Nelís Soto-Ramírez, Hongmei Zhang

**Affiliations:** 1 Division of Epidemiology, Biostatistics and Environmental Health, School of Public Health, University of Memphis, Memphis, TN 38119 USA; 2 College of Social Work, University of South Carolina, Columbia, SC 29208 USA

**Keywords:** Infant feeding, Breastfeeding, Formula feeding, Solid food feeding, Pumping and feeding, United States of America

## Abstract

**Background:**

Infant feeding may consist of direct breastfeeding (DBF), pumping and bottle feeding (P&F), formula feeding (FF), solid food feeding (SFF), and any combination. An accurate evaluation of infant feeding requires descriptions of different patterns, consistency, and transition over time.

**Methods:**

In United States of America, the Infant Feeding Practice Study II collected information on the mode of feeding on nine occasions in 12 months. We focused on the first 6 months with six feeding occasions. To determine the longitudinal patterns of feeding the latent class transition analyses was applied and assessed the transition probabilities between these classes over time.

**Results:**

Over 6 months, 1899 mothers provided feeding information. In month 1 the largest latent class is FF (32.9%) followed by DBF (23.8%). In month 2, a substantial proportion of the FF class included SFF; which increases over time. A not allocated class, due to missing information was identified in months 1-3, transitions to SFF starting in month 4 (8.9%). In month 1, two mixed patterns exist: DBF and P&F combined with FF (13.9%) and DBF combined with P&F (18.7%). The triple combination of DBF, P&F, and FF (13.9%) became FF in month 2 (transition probability: 24.8%), and DBF in combination with P&F (transition probability: 49.1%). The pattern of DBF combined with P&F is relatively stable until month 4, when at least 50% of these infants receive solid food. Only 23-26% of the infants receive direct breastfeeding (DBF) in months 1-4, in month 5-6 SFF is added. Mothers who used FF were less educated and employed fulltime. Mothers who smoke and not residing in the west of the United States were also more likely to practice formula feeding.

**Conclusion:**

Infant feeding is complex. Breastfeeding is not predominant and we additionally considered the mixed patterns of feeding. To facilitate direct breastfeeding, a substantial increase in the duration of maternal leave is necessary in the United States.

## Background

Food that mothers feed their babies is paramount for the development of the child. Inappropriate infant feeding practices can predispose children to wheezing, obesity, and lifelong health problems [1, 2]. Therefore, optimal feeding practices are critical to provide appropriate nutrition during infancy [3–9]. Infant feeding practices can include breast milk feeding, formula feeding, and supplementary feeding. The American Academy of Pediatrics (AAP) Section on Breastfeeding, American College of Obstetricians and Gynecologists, American Academy of Family Physicians (AAFP), Academy of Breastfeeding Medicine, World Health Organization (WHO), United Nations Children’s Fund, and many other health organizations recommend exclusive breastfeeding for the first 6 months of life [10, 11]. Exclusive breastfeeding includes breast milk with no solids or other liquid foods except vitamin/mineral supplements or medication [12–14].

The distinction of exclusive and non-exclusive breastfeeding does not consider the actual mode of feeding. In addition, it assumes that the quality of breast milk is independent of whether it is provided direct at the breast (direct breastfeeding [DBF]) or feeding of pumped breast milk in a bottle (indirect breastfeeding or pumping and feeding [P&F]). However, direct breastfeeding is considered being superior since pumped milk may not provide the same protective qualities of milk direct from the breast and does not require the exercise of sucking [15, 16]. If DBF is not feasible, pumping and feeding is considered an alternative [17, 18]. In addition to breastfeeding, supplementary feeding types consist of formula feeding (FF) and feeding of complementary foods such as liquids food and solid foods feeding (SFF) [11, 19, 20].

Despite the strong endorsement for breastfeeding, most infants in the United States (US) are fed some formula by the time they are 2 months of age. Complementary feeding is considered to increase the risk for obesity and diabetes later in life [21–25]. In addition, introduction of complementary food before 6 months decreases breast milk consumption [26–29], increases the risk of choking [30], allergic reaction [31], and may not be favorite for the cognitive development of the child [32, 33]. Therefore, the WHO recommends that infants should be gradually introduced to complementary foods around 6 months of age [34]. Regardless of benefits and recommendations, approximately 85% of mothers globally are not in compliance [26].

However, critical information on how infant feeding develops during the first 6 months is missing. Such information would provide necessary information for public health, for instance, which combinations of infant feeding exist, which infant feeding modes are likely to be maintained, and which more likely transition to other modes. In addition, information on combined feeding modes will provide a more accurate exposure assessment to infant food than the calculation of duration of breastfeeding or of exclusive breastfeeding. However, these two makers are predominately used in attempts to assess health benefits and risks of infant feeding.

To the best of our knowledge, the Infant Feeding Practice Study II (IFPS II), conducted by the Food and Drug Administration (FDA) and the Centers of Disease Control (CDC), is the only study that collected longitudinal feeding data during the first year [35]. Our focus is on the first 6 months for two reasons. First, breastfeeding is still prevalent in the first 6 months and it is possible to observe who opts out of breastfeeding. Second, limitations of the statistical analyses of complex patterns over time forced us to limit combinations of feeding modes and respective months of observations. We analyzed the unique IFPS II data set to describe typical combinations of feeding practices and their changes in the course of the first 6 months.

The primary aim was to identify longitudinal patterns of different and combined modes of breastfeeding, which we call breastfeeding trajectories. For this purpose, we identified patterns of co-existing feeding modes, their persistence (homotypic continuity), and transitions among practices (heterotypic continuity) [36]. Second, we were interested to characterize factors that influence different feeding modes.

## Methods

### Study design

The IFPS II collected information from mothers from May 2005 through June 2007 [35]. The sampling frame for the IFPS II was a nationally distributed consumer opinion panel of 500,000 households. IFPS II provides longitudinal data starting with a prenatal questionnaire; a short telephone interview near the time of the infant’s birth; a neonatal questionnaire sent when the infant was about 1 month old; and 9 questionnaires until the infant was 12 months old. The self-administered questionnaires included data on infant feeding, infant health, care, life style factors, employment, and demographics [35]. A total of 2988 participants provided any information about infant feeding; the sample with complete infant feeding information in the first 6 months included 1899 women.

### Questionnaire data

The mode of infant feeding variable in the first 6 months was assembled from four questions repeatedly asked in the first six post-natal questionnaires (Table 1). Direct breastfeeding DBF and P&F were ascertained by the questions “Does your baby usually feed from both breasts at each feeding? (Question N56)” (yes, no, “Baby is only fed pumped milk”); “Does your baby usually let go of the breast him or herself (Question N57)” (yes, both breast; yes, first breast only; yes, second breast only; no). These two questions combined with the following query “How many times in the past seven days was your baby fed expressed or pumped milk to drink (Question N61)” was used to distinguish DBF and P&F. The question “In the past 7 days, how often your baby was fed each food listed below? (Question N40a and N40b)” (breast milk, formula) was used to determine whether the infant was fed breastmilk or formula. Table 1 shows the definition and the respective number of women for each response in the first month. In addition, we classified feeding of complementary foods into solid food (baby cereal, other cereals and starches, vegetables, French fries, fruits, meat, fish or shellfish, eggs, peanut foods, dairy foods, and soy foods) and liquid food (cow’s and other milk, fruit and vegetable juice, and sweet drinks) by the question “How often your baby fed each food list below?” [37]. The information on solid food feeding (SFF) was inquired independent of other feeding modes. Questionnaires were not always completed and returned at the required age of the infants. All time-dependent variables (here: infant feeding) were corrected for the actual age of the completion date if the completion date was past the accepted interval listed on the questionnaire [37].

**Table 1:** Definition of infant feeding modes in the Infant Feeding Practice Study (IFPSII) questionnaire^a^ (*n* = 1899 with valid answers)

Mode of feeding	Does your baby usually feed from both breasts at each feeding? (Question N56)^b^	Does your baby usually let go of the breast him or herself? (Question N57)	How many times in the past 7 days was your baby fed expressed or pumped breast milk to drink? (Question N61)	In the past 7 days, how often was your baby fed each food listed below? (Question N40a): Breastmilk	In the past 7 days, how often was your baby fed each food listed below? (Question N40b): Formula	Month 1	
						*n*	Total
Formula feeding (FF)	Instructed to skip if not breastfeeding		Instructed to skip	= 0	≥ 1	508	508
			0		≥ 1	0	
Pumping & feeding (P&F) combined with formula feeding (FF)	Valid answer	Missing	≥ 1	> 0	≥ 1	61	70
	Valid answer	Missing	0		≥ 1	9	
Pumping and feeding (P&F)	Baby is fed only pumped milk	Skipped after *n* 56	≥ 1	> 0	0	18	18
Pumping & feeding (P&F) combined with direct breastfeeding (DBF)	Valid answer	No	≥ 1	> 0	0	53	341
	Valid answer	Yes	≥ 1		0	285	
	Valid answer	Missing	≥ 1		0	3	
Direct breastfeeding (DBF)	Valid answer	No	0	> 0	0	56	457
	Valid answer	Yes	0		0	398	
	Valid answer	Missing	0		0	3	
Direct breastfeeding (DBF) and formula feeding (FF)	Valid answer	No	0	> 0	≥ 1	42	233
	Valid answer	Yes	0		≥ 1	190	
	Valid answer	Missing	0		≥ 1	1	
Mixed: direct breastfeeding (DBF), Pumping & feeding (P&F), formula feeding (FF)	Valid answer	Yes	≥ 1	> 0	≥ 1	224	272
	Valid answer	Missing	≥ 1		≥ 1	3	
	Valid answer	No	≥ 1	> 0	≥ 1	45	

^a^ The questionnaires used in the IFPSII can be assessed at https://www.cdc.gov/breastfeeding/data/ifps/questionnaires.htm

^b^ The numbers (e.g., *n* 56) refer to the numbering of the questions in the publicly available data-set

Maternal age during pregnancy was categorized (18-24, 25-34, 35-43 years). During the prenatal survey, information on maternal race (White, Black, Asian/Pacific Islander, and a category for other races) and Spanish ethnicity (Mexican, Puerto Rican, Cuban, other Spanish/Hispanic) was collected. The two variables were grouped into the categories of White, Black, Hispanic, and other races. In addition, information on region of residence (Northwest, Midwest, South, and West) and level of education (grouped into high school graduate or less and college graduate) was ascertained. Percent of poverty level was determined by reported income (reference = 185%, 185-349%, > 350%). Maternal employment was based on the answer of question “Did you work for pay any time during the past 4 weeks?”, fulltime, part-time, unemployed, homemaker. The prenatal occupational title was assessed based on maternal reports of her type of work. It was grouped as follows: 1) administrative (professional specialty, executive, administrative and managerial, administrative support, including clerical); 2) production and farming (precision production, craft and repair, operator, fabricator and laborer, technician and related support, farming, forestry and fishing); 3) sales and services; 4) not employed. The prenatal smoking status (current average daily cigarettes smoked) was categorized into yes and no. The marital status was grouped into married and unmarried (widowed, divorced, separated, never married). Before delivery, the mother was also asked whether she was enrolled in a health insurance or health care plan (yes, no) and whether she participated in the special supplemental nutrition program for Women, Infants, and Children (WIC). In the neonatal survey, the number of other babies the mother had was used to categorize parity (0, 1, 2, 3, 4 and more).

### Statistical analysis- feeding trajectories

To identify patterns of various combinations of infant feeding based on information on direct breastfeeding (DBF), indirect breastfeeding or pumping and feeding or breastmilk [P&F], formula feeding (FF), and solid food feeding (SFF), we applied latent class transition analyses (LTA). LTA simultaneously discovers latent classes of feeding practices then assesses transition probabilities between these latent feeding classes over the course of the investigation [38–41]. The LTA program designed for Statistical Analysis System (SAS, Version 9.4) [42, 43] estimates three types of parameters: (1) probabilities of response to items describing the latent classes, conditional on latent status and time; (2) probability of membership in each latent status at each time point (1-6 months); and (3) conditional transition probabilities from one latent class at time t to another class at time t + 1. Missing data were handled with a full-information maximum likelihood (FIML) technique. The different infant feeding modes (DBF, P&F, FF, and SFF) were response items and were used to describe latent classes of infant feeding in each of the 6 months. A latent class was labeled DBF, P&F, FF, and or SFF, when 50% or more of its member reported the respective response item. The optimal number of classes was determined using G^2^, which measures the degree of agreement between the predicted and observed response proportions in each latent class, and the Akaike’s information criterion, which evaluates the penalized likelihood of a particular classification and transition pattern. When the addition of classes provides no essential improvement of fit relative to the drop in degrees of freedom, then the model fit is best [39]. Models with different numbers of latent classes were tested, starting with two latent classes and increasing sequentially to a 9-latent status model. Since 40 combinations of classes and times (months) were the maximum with four response items (DBF, P&F, FF, and SFF), we tested the appropriateness of using more classes (up to nine) by restricting the number of response items (DBF, P&F, and FF), ignoring feeding of solid foods.

### Effects of maternal characteristics on infant feeding modes

To gain understanding, which maternal factors influence the mode of infant feeding (DBF, P&F FF, and SFF) we used the repeated information on infant feeding collected from the mother in the course of the first 6 months. These repeated measurements are correlated over time. Hence, we applied generalized estimation equation (GEE) analyses that take the within-mother correlations into account [44]. With the exemption of the WIC status, maternal characteristics were collected before or shortly after birth and changes in the first 6 months are not considered. To describe the importance of maternal characteristics, we estimated risk ratios (RR) and their 95% confidence intervals (95% CI). Statistically we controlled to age of the infant. The twelve maternal risk factors include maternal age, race/ethnicity, region of residence, education, income, employment status, occupational title, smoking, health insurance, participation in the special supplemental Nutrition Program for Women, Infants, and Children (WIC), marital status, and parity were considered as time-independent variables. To adjust for multiple testing (12 risk factors, 23 levels) in each of four modes of infant feeding, we controlled for false-discovery rate (FDR) preventing a large proportion of false positives [45]. The Statistical Analysis Systems, version 9.4 (SAS institute, Inc., Cary, NC) software package was used to analyze the data.

## Results

Complete feeding information for the first 6 months was provided by 1899 participants: 61.7% of participants were between 25 and 34 years old, 84.8% of them were white, 78.1% of the mothers had College education of more, and 78.8% were married (Table 2). There was no statistical significant difference between the sample that provided any breastfeeding information (*n* = 2988) and the sample with complete data in the first 6 months (*n* = 1899) used in this analysis.

**Table 2:** Characteristics of mothers with six follow-ups compared to mothers which any feeding information

	Total sample with any infant feeding information		Sample with complete feeding information in the first 6 months		*p* - values, comparing the two samples (Chi^2^-square tests)
	*n* = 2988	%	*n* = 1899	%	
Age					
18-24	686	23.0	425	22.5	0.82
25-34	1835	61.6	1166	61.7	
35-43	456	15.3	300	15.9	
Missing	11		8		
Race/Ethnicity					
White	2458	84.6	1570	84.8	0.62
Black	135	4.7	75	4.1	
Hispanic	179	6.2	110	5.9	
Other	134	4.6	96	5.2	
Missing	82		48		
Region					
Northwest	516	17.3	342	18.0	0.86
Midwest	903	30.2	576	30.3	
South	967	32.4	596	31.4	
West	602	20.2	385	20.3	
Education					
HS graduate or less	574	23.2	350	21.9	0.36
College graduate	1903	76.8	1245	78.1	
Missing	511		304		
Income (% of poverty)					
< 185%	1247	41.7	834	42.9	0.71
185%-349%	1072	35.9	681	35.0	
≥350%	669	22.4	429	22.1	
Employed					
Full-time	1958	77.0	1267	77.2	0.91
Part-time	327	12.9	214	13.0	
Unemployed	259	10.2	161	9.8	
Missing	444		257		
Area of employment					
Administrative, etc.	844	36.4	530	35.4	0.65
Operator, Farming	130	5.6	98	6.6	
Sales, Service	293	12.6	186	12.4	
Not employed	1051	45.3	682	45.6	
Missing	670		403		
Smoking status					
Yes	306	10.2	188	9.9	0.69
No	2682	89.8	1711	90.1	
Health insurance					
Yes	2847	95.4	1812	95.5	0.81
No	138	4.6	85	4.5	
Missing	3		2		
WIC status					
Yes	933	31.3	574	30.3	0.45
No	2050	68.7	1323	69.7	
Missing	5		2		
Marital Status					
Yes	2190	79.2	1398	78.8	0.73
No	576	20.8	377	21.2	
Missing	222		124		
Parity					
0	849	28.4	560	29.5	
1	1187	39.7	737	38.8	
2 and higher	876	29.32	555	29.23	0.70
Missing	76		47		

The single and combined modes of feeding identified from the questionnaires (Table 1) are presented in Table 3 for the first 6 months. Solid food feeding (SFF) was determined independently of other infant feeding practices, therefore in Table 3 the sum of all feeding modes is larger than 100%. The proportion of women who directly breastfed their offspring (DBF) was declining from 24.1% in month one to 20.9% in month six. Isolated pumping and feeding (P&F) was detected in only 1% of the mothers. Also the proportion of women practicing P&F combined with DBF is diminishing from 18.0% in month one to 13.0% in month six. Formula feeding increases and so does feeding of solid food. At age 2 months, 11.9% of the children received supplemental solid food, but already 41.2% in months 4 and 73.1% in month five.

**Table 3:** Proportion (in %) of different modes of feedings in the first 6 months (*n* = 1899)

Age of infant	Month 1	Month 2	Month 3	Month 4	Month 5	Month 6
Mode of feeding	% (*n*)	% (*n*)	% (*n*)	% (*n*)	% (*n*)	% (*n*)
Formula feeding (FF)	26.8 (508)	34.2 (428)	37.6 (545)	42.5 (549)	45.0 (599)	48.0 (619)
Pumping & feeding (P&F) combined with formula feeding (FF)	3.7 (70)	2.2 (27)	1.8 (26)	1.6 (21)	2.0 (26)	1.4 (18)
Pumping & feeding (P&F)	0.95 (18)	1.2 (15)	0.7 (10)	0.7 (9)	0.6 (8)	0.8 (10)
Pumping & feeding (P&F) combined with direct breastfeeding (DBF)	18.0 (341)	20.9 (261)	20.9 (303)	17.7 (229)	15.5 (206)	13.0 (167)
Direct breastfeeding (DBF)	24.1 (457)	19.8 (248)	19.9 (288)	19.9 (257)	20.3 (270)	20.9 (270)
Direct breastfeeding (DBF) and formula feeding (FF)	12.3 (233)	10.5 (131)	9.4 (136)	8.0 (103)	8.8 (117)	9.3 (120)
Mixed: direct breast feeding (DBF), Pumping & feeding (P&F), formula feeding (FF)	14.3 (272)	11.3 (142)	9.7 (141)	9.6 (124)	8.0 (106)	6.7 (86)
Solid food feeding (SFF) ^a^	5.8 (112)	11.9 (618)	19.1 (425)	41.2 (561)	73.1 (540)	91.4 (593)

^a^ Solid food feeding (SFF) was queried independently from other feeding modes. Hence, in this table there is no overlap of solid food with other feeding and all proportions do not add up to 100%. The proportions provided in this table also do not fit completely with the proportion in Fig. 1, which proportions add up to 100% each month of observation

**Fig. 1:**
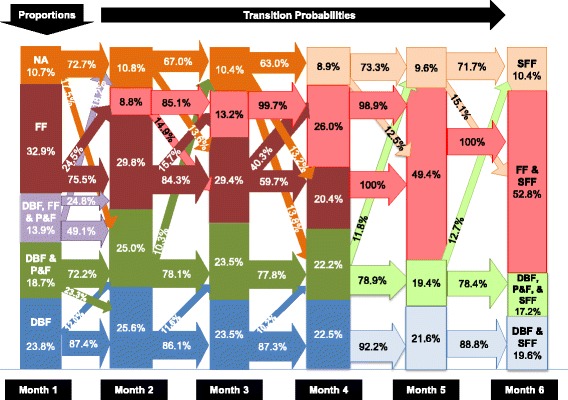
Proportion and transition probabilities of infant feeding practice (*n* = 1899) in the US. Figure shows the development of infant feeding in the first 6 months. Direct breastfeeding (DBF - blue) was practiced in the first month by 23.8% of the women. In the second month, a few women (23.3% from DBF and pumping and feeding [P&F] group) moved to the DBF group. In month 5, nearly all infants from the DBF group were introduced to solid food feeding (SFF). The same pattern is seen for the mix of direct DBF and P&F (green). The group of women with a mixed feeding combination (DBF, P&F, FF - violet) dissolved into either formula feeding (FF) or the combination of DBF + P&F in month 2. Formula-feed (FF) infants (32.9%, dark red) experience increasing introduction of solid food (SF-pink) from month 2-5

Results of Latent Class Transition (LTA) analyses indicate that a classification into six classes fits the data structure best in the first 6 months. To ease the understanding of patterns, we present the combinations of feeding modes detected in each month as vertical columns (six columns) and the changes over time (transition probabilities) as arrows between the classes (Fig. 1). We focus on transition probabilities larger than 10%. The proportions shown in the columns indicate the prevalence of specific latent feeding classes. The transition probabilities (arrows between the columns) display the proportion of transitions between latent classes (conditional transition probabilities). The thickness of the arrows stands for magnitude of the transition probability.

In month one, the largest latent class is formula feeding only (FF, 32.9%) followed by direct breastfeeding only (DBF, 23.8%). There are two latent classes with combinations: DBF, P&F combined with FF (13.9%) as well as DBF combined with P&F (18.7%). In months 1-3, about 10-11% of the feeding modes could not be allocated to one latent class (not allocated, NA). Some questionnaire information was not available for single months (intermittent missing values), but available before or after that month, which is can be compensated by the latent class transition analysis. However, the ‘not allocated’ status was due to repeated missing information in months one to three.

Inspecting the development (Fig. 1), is it obvious that the proportion of women who practice only formula feeding (FF) declines from months one to four, from 32.9% to 20.4%. However, starting in month two, a large proportion of the FF class adds solid food feeding (SFF) as infant feeding (transition probability: 24.5%). Hence, the proportion of FF combined with SFF increases over time. At month five, the whole FF class has submerged into the latent class of “FF combined with SFF”. Surprisingly, the not allocated class (NA), identified in months one to three, shows that most of the infants in this group (prevalence 9-10%) were predominately fed solid food starting in month four. Small proportions of the NA class move between month two to five to formula feeding (transition probabilities: 13.6% and 13.2%) and the combination of DBF and P&F (transition probabilities: 17.5% and 13.8%).

The triple combination of DBF, P&F combined with FF in month one (13.9%) consists only briefly and dissolves in month two into NA (transition probability: 18.2%), FF (transition probability: 24.8%), and the class “DBF combined with P&F” (transition probability: 49.1%). The latent class of “DBF combined with P&F” is relatively stable until month four (Fig. 1), when at least 50% of infants in this class received solid food (DBF and P&F in combination with SF). The same development is seen for the “DBF only” class; 23% to 26% of the infants fall into this class during months one to four. In months five to six, solid food (SF) is added to this latent class (DBF in combination with SF).

Results of latent class analyses not including solid food feeding (SFF) show similar patterns (data not shown) and are used to determine if more than six classes of DBF, P&F, and FF were needed.

When analyzing maternal characteristics that are associated with repeated measurement of infant feeding modes (DBF, P&F, FF, and SF) in the first 6 months (Table 4), we controlled for false-discovery rate (FDR) to minimize false positive findings. Regarding direct breastfeeding (DBF), only maternal smoking and the supplemental nutrition program for Women, Infants, and Children showed associations that survived the Bonferroni correction: mothers who smoke were 45% less likely to directly breastfeed and had 60% increase in formula use. Most differences were seen for formula feeding (FF). Interestingly, compared to the West of the United States, women in all other regions were about 40% more likely to use formula feeding (*p* - values between 0.0002 and 0.0003). Regarding education, “High school or less” was associated with higher risk ratios of feeding formula (*p* = 0.0002). Women with fulltime employment were 25% more likely to use formula feeding, against that, not employed women were 20% less likely to use formula (Table 4).

**Table 4:** Maternal characteristics as risk factors for different modes of feeding

Risk Factors		Direct breastfeeding 6617 observations in 1364mothers		Feeding of pumped breast milk 6617 observations in 1364 mothers		Formula feeding 6617 observations in 1364 mothers		Feeding of solid foods 6725 observations in 1899 mothers	
		RR (95% CI)	*p*-value^§^	RR (95% CI)	*p*-value^§^	RR (95% CI)	*p*-value^§^	RR (95% CI)	*p*-value^§^
Age (years)	18-24	0.82 (0.70-0.96)	0.049	0.83 (0.66-1.01)	0.20	1.19 (1.07-1.33)	0.005	1.00 (0.92-1.11)	0.93
(reference: 25-34)	> 34	1.03 (0.93-1.15)	0.70	1.01 (0.87-1.17)	0.97	0.99 (0.87-1.13)	0.96	1.05 (0.97-1.15)	0.47
Race	Black	0.84 (0.61-1.16)	0.43	1.01 (0.70-1.48)	0.97	1.02 (0.87-1.19)	0.93	1.12 (0.93-1.36)	0.47
White (ref)	Hispanic	0.85 (0.69-1.04)	0.22	1.17 (0.94-1.46)	0.29	1.14 (1.01-1.29)	0.07	0.95 (0.83-1.08)	0.56
	Other	1.0 (0.84-1.19)	0.98	1.00 (0.78-1.29)	0.97	0.99 (0.84-1.19)	0.99	0.96 (0.82-1.12)	0.68
Region	Northeast	0.78 (0.68-0.90)	0.007	0.91 (0.75-1.11)	0.50	1.40 (1.19-1.64)	0.0003	1.12 (1.02-1.23)	0.11
West (reference)	Midwest	0.90 (0.81-1.01)	0.17	1.06 (0.91-1.25)	0.60	1.37 (1.20-1.59)	0.0002	1.09 (1.00-1.19)	0.24
	South	0.83 (0.73-0.92)	0.007	0.99 (0.84-1.17)	0.97	1.35 (1.17-1.55)	0.0003	1.15 (1.05-1.26)	0.02
Education	High school or less	0.81 (0.71-0.94)	0.034	0.80 (0.65-0.97)	0.15	1.33 (1.21-1.48)	0.0002	1.17 (1.08-1.28)	0.003
College (reference)									
Poverty (% of poverty level)	185%-349%	1.02 (0.92-1.14)	0.75	1.21 (1.02-1.43)	0.14	0.95 (0.85-1.05)	0.41	1.05 (0.97-1.14)	0.47
	> 350%	0.98 (0.86-1.12)	0.81	1.30 (1.08-1.58)	0.12	1.01 (0.90-1.15)	0.93	0.93 (0.84-1.03)	0.47
(< 185%: reference)									
Employment status	Full-time	1.66 (0.89-3.11)	0.22	1.65 (0.94-2.88)	0.20	1.25 (1.11-1.41)	0.0007	0.99 (0.74-1.31)	0.97
(Homemaker: reference)	Part-time	1.74 (0.93-3.28)	0.20	1.60 (0.90-2.84)	0.24	1.13 (0.98-1.30)	0.16	0.91 (0.68-1.22)	0.65
	Unemployed	0.97 (0.80-1.19)	0.81	1.21 (0.90-1.63)	0.34	1.25 (1.09-1.44)	0.0046	1.00 (0.85-1.18)	0.98
Employment^a^ (Administrative work: reference)	Production /Farm	1.18 (1.01-1.38)	0.12	0.97 (0.79-1.20)	0.97	0.87 (0.72-1.05)	0.19	0.94 (0.83-1.08)	0.56
	Sales, Service	0.93 (0.79-1.10)	0.54	0.82 (0.66-0.99)	0.20	1.02 (0.90-1.17)	0.73	1.04 (0.95-1.15)	0.56
	not employed	1.91 (1.03-3.55)	0.12	1.21 (0.70-2.09)	0.62	0.80 (0.72-0.88)	0.002	0.86 (0.65-1.14)	0.47
Smoking status^b^	Yes	0.55 (0.41-0.73)	0.0008	0.75 (0.55-1.03)	0.20	1.60 (1.43-1.70)	0.002	1.20 (1.08-1.33)	0.009
Health insurance^c^	No	0.93 (0.77-1.13)	0.60	0.85 (0.70-1.03)	0.5	1.19 (0.94-1.51)	0.21	1.11 (0.93-1.31)	0.47
WIC status^b^	Yes	0.84 (0.74-0.96)	0.047	0.81 (0.62-1.02)	0.20	1.20 (1.08-1.33)	0.053	1.09 (1.01-1.19)	0.18
Marital Status^c^	No	0.89 (0.76-1.05)	0.30	0.86 (0.70-1.04)	0.26	1.29 (0.97-1.73)	0.0032	1.05 (0.96-1.15)	0.47
Parity (2 or more children: reference)	0	0.92 (0.81-1.06)	0.36	1.27 (1.06-1.53)	0.12	1.23 (1.08-1.42)	0.007	1.04 (0.94-1.14)	0.60
	1	0.94 (0.85-1.03)	0.37	1.11(0.95-1.30)	0.32	1.17 (1.04-1.33)	0.21	1.05 (0.97-1.13)	0.47

^a^Employment: 1) production and farming (precision production, craft and repair, operator, fabricator and laborer, technician and related support, farming, forestry and fishing); 2) sales and services; 3) not employed; reference: administrative (professional specialty, executive, administrative and managerial, administrative support, including clerical)

§*p -* value after adjustment for false discovery rate

^b^No is the reference

^c^Yes is the reference

Younger women (age 18-24) were less likely to direct breastfeed their infant (RR = 0.82, 95% CI 0.70, 0.96) compared to the reference (25-34 years of age), and more likely to formula feed (RR = 1.19, 95% CI 1.07, 1.33), see Table 4. Interestingly, compared to the West, women residing in the Northeast and the South of the United States showed a lower proportion of DBF (RR = 0.78 and 0.83, respectively). Again, compared to the West, women in all other regions were about 40% more likely to use formula feeding. Regarding education, lower education such as “High school or less” was associated with higher risk ratios of both, feeding formula and solid food.

The employment status showed a significant increased risk of formula feeding for fulltime working and women with an unemployment status. Against that, being not-employed shows a 20% lower probability of formula feeding (Table 4). Maternal smoking was related to a 45% reduction in direct breastfeeding, a 60% increase in formula and a 20% increase in solid food feeding. Participation in the supplemental nutrition program for Women, Infants, and Children (WIC) was related to a 16% lower proportion of direct breastfeeding (DBF). Infants from unmarried women had a 29% higher risk of formula feeding; first-born infant (parity = 0) had 23% higher risk of formula feeding.

## Discussion

Allowing for six latent classes, we identified nine trajectories of infant feeding in the course of 6 months (Fig. 1). These were (1) formula feeding, (2) solid food feeding, (3) formula combined with solid food, (4) a combination of direct feeding at the breast, pumping and feeding, and formula feeding, (5) a mix of direct breastfeeding with pumping and feeding, (6) which was then in months five supplemented with solid food, (7) exclusive direct breastfeeding, (8) also later supplemented with solid food, and (9) a short trajectory with missing information in months one to three. In the first month, most mothers fed their infants at least partially with breast milk (DBF) 23.8%, pumping and feeding (P&F) 18.7%, DBF, P&F and formula feeding (FF) 13.9%), total of 56.4%. However, by the fourth month of age, formula and solid food predominates (55.3%): formula feeding (FF, 26.0%), solid food feeding (SFF, 8.9%), and the combination of FF and SFF (20.4%). In month six, only 36.8% of the infants experience some direct breastfeeding combined with pumping and feeding and solid food.

Regarding maternal characteristics related to different feeding modes, results of previous studies showed a similar description of infant feeding practices in U.S. and demonstrated that many mothers introduced solid foods earlier than recommended [46–48]. Compared to the west region in the United States, women in the Northeast, Midwest, and South were about 40% more likely to use formula feeding (FF). Higher education, fulltime work, unemployment, and smoking during pregnancy were also associated with formula feeding. Other studies have presented similar effects of maternal characteristic on infant feeding practices [49–55].

Past research also showed, describing each month separately, two to three coexisting feeding modes [56–60] or showed changes of different formula brands over time [61]. However, this is the first study that comprehensively identifies the course of different feeding modes over time. These feeding trajectories provide a more accurate exposure assessment of infants to different food stuff than a simple calculation of breastfeeding duration.

The strengths of this study include its prospective design, detailed information about infant feeding practices and a large sample size. In addition, the information was collected each month for the preceding one- or two-week period, which minimizes recall bias [62]. However, this study had some limitations. The sample, although well distributed throughout the U.S., was not representative of the U.S. population. Most women in the sample were white, higher educated, employed, had a higher socioeconomic status, and were not in the WIC program. Furthermore, our sample only included mothers who were willing to participate, and all data were self-reported by the mother, which may create an unknown bias. In addition, in order to analyze infant feeding trajectories from months one to six, we had to focus on mothers with a more complete report of infant feeding practices; however, our sample was not different from the sample with any infant feeding information (Table 2). Finally, with the exception of the WIC status, maternal characteristics were collected only once shortly before or after birth. Their effects were assessed for the following 6 month of infant feeding. Hence, the data did not allow us, to estimate the role of changes (e.g., employment status) in the first 6 months of infant feeding.

In our study, we found a much lower proportion of breastfeeding and higher proportion of solid foods introduced early than recommended by WHO/UNICEF or the American Academy of Pediatrics [3, 7, 63], suggesting a low proportion of exclusive breastfeeding in the first 6 months. Also the proportion of mixed feeding of different food stuff (direct breastfeeding, pumping and feeding, combined with formula or solid food feeding) is of concern, since the infant is early exposed to a large number of foreign substances, which may trigger an allergenic reaction [2]. One reason of combining different feeding modes seems to be related to the status of the mother: fulltime work, not un-employed, and smoking during pregnancy were related to formula feeding, mostly related to the burden of fulfilling multiple daily tasks. Women’s work was identified as a leading motive for not breastfeeding and early weaning. Short maternity leave (< 6 weeks) is considered to result in a four-time increase in the risk of not establishing or early cessation of breastfeeding [63]. Analyses of 172 different nations showed that 69% of all countries provide paid breastfeeding leave for 6 months or more [64] but not the United States of America, which however is a critical measure to increase the proportion of mothers who have a chance to breastfeed [65].

## Conclusions

Infant feeding in the first 6 months show complex trajectories of mixed feeding modes. Breastfeeding is not the main mode of infant feeding. To appropriately assess health risks of infant feeding, we need to take into account mixed or combined patterns and not only the duration of breastfeeding. Mothers who used direct breastfeeding or pumping and feeding were more likely to be higher educated and part-time working. Fulltime employment and non-married mothers had a higher risk of formula feeding. Mothers who initiated direct breastfeeding in the first month were more likely to stay with this choice compared to the mothers who started with mixed feeding modes. To enable more women to practice direct breastfeeding, a substantial increase in the duration of maternal leave is necessary in the United States, comparable to 119 other countries in the world.

## Abbreviations

95% CI: 95% confidence interval for the RR
DBF: Direct breastfeeding
FF: Formula feeding
NA: Not allocated pattern of feeding
P&F: Pumping and feeding of breast milk
RR: Risk ratio
SFF: Solid food feeding
